# Implementation strategies, facilitators, and barriers to scaling up and sustaining task-sharing in family planning: a protocol for a mixed-methods systematic review

**DOI:** 10.1186/s13643-023-02356-5

**Published:** 2023-10-07

**Authors:** Adeniyi Kolade Aderoba, Rita Kabra, James Njogu Kiarie

**Affiliations:** 1 Centre for Tropical Medicine and Global Health, Nuffield Department of Medicine, University of Oxford, Oxford, UK; 2 Centre for Population Health and Interdisciplinary Research, HealthMATE360, Box 603, Ondo Town, Ondo State Nigeria; 3 UNDP/UNFPA/UNICEF/WHO/World Bank Special Programme of Research, Development and Research Training in Human Reproduction, World Health Organization Headquarters, Geneva, Switzerland

**Keywords:** Implementation strategies, Facilitators, Barriers, Outcome, Scale-up, Sustainability, Task-shifting, Task-sharing, Family planning, Contraception, Systematic review

## Abstract

**Background:**

Ensuring access to quality family planning (FP) services is fundamental to achieving the Sustainable Development Goals (SDG) targets 3.1, 3.7, and 5.6, including universal access to reproductive health services. However, barriers such as health workforce shortages and restrictive policies on the role of mid and lower-level health workforce cadres limit access to contraceptives and FP in many settings.

Workforce reorganization makes more efficient use of human resources. Consequently, the World Health Organization (WHO) recommends task-sharing for FP by different cadres. Evidence on the implementation strategies, facilitators, and barriers to scaling up and sustaining task-sharing could inform financing, implementation approaches, and technical assistance of national and global FP task-sharing programs. Therefore, this study aims to describe and assess the quality of the evidence on implementation strategies, facilitators, and barriers to scaling up and sustaining task-sharing in FP and the outcome of the scale-up/sustainability interventions.

**Methods:**

This systematic review protocol was developed using relevant guidelines, including the Preferred Reporting Items for Systematic Review and Meta-Analysis (PRISMA) Protocols (PRISMA-P). A search of five databases, namely CINAHL (EBSCOhost), EMBASE (OvidSP), Global Health (OvidSP), MEDLINE (OvidSP), and Scopus (www.scopus.com), and gray literature resources will be conducted. Two independent reviewers will screen and select studies, assess their quality using the “Mixed Methods Appraisal Tool,” and extract data from eligible studies. Publications or articles are eligible if they report implementation strategies, facilitators, or barriers to scaling up/sustainability of task-sharing in FP/contraception or the outcomes of the scale-up/sustainability interventions. A convergent synthesis that integrates qualitative, quantitative, descriptive, and mixed-methods data into one dataset will be used for analysis based on an a priori framework—the Cochrane Effective Practice and Organization of Care (EPOC) taxonomy of the health system framework. Two independent reviewers will assess the quality of evidence using the GRADE-CERQual guideline.

**Discussion:**

To our knowledge, this systematic review of implementation strategies, facilitators, and barriers to scaling up and sustaining task-sharing in family planning is the first in this area. Our rigorous methodology based on up-to-date guidelines can help generate relevant recommendations to support interventions to scale up and sustain task-sharing in family planning.

**Systematic review registration:**

PROSPERO CRD42022339885.

## Introduction

Ensuring access to quality family planning (FP) services is fundamental to upholding human rights and achieving the Sustainable Development Goals (SDG) targets 3.1, 3.7, and 5.6, including universal access to reproductive health services. Among 1.6 billion women of reproductive age living in low- and middle-income countries (LMICs), 923 million want to avoid pregnancy [1]. However, about one in every four of these women have an unmet need for FP that threatens their health and lives and negatively impacts their families [1]. If these unmet needs were addressed and women received optimal FP services, unintended pregnancies would be reduced by 68%, unsafe abortions by 72%, and maternal deaths by 62% [1]. Contraceptives are relatively inexpensive and cost-effective interventions. Still, barriers such as health workforce shortages and restrictive policies on the role of mid and lower-level health workforce cadres limit access to FP in many settings [2].

High-impact practices in FP (HIPs) are curated, up-to-date promising scalable interventions across settings that can strengthen FP programs [3]. These HIPs include expanding contraceptive access and uptake by rationally moving some FP-skilled health personnel tasks to less-specialized cadres such as community health workers [4]. Consequently, workforce reorganization makes more efficient use of human resources.

To reorganize the health workforce and make more efficient use of human resources, the World Health Organization (WHO) recommends task-sharing for FP by different cadres [5, 6]. Task-sharing refers to expanding health cadres who can appropriately deliver health services, i.e., all or components of a clinical task hitherto restricted to higher-level cadres are shared with designated cadres of health workers, but not the removal, delegation, or rational distribution from one cadre to another, termed task shifting [2, 7]. The WHO 2017 guidelines on task-sharing for FP [2] recommended that community health workers have the necessary skills to educate, counsel, and provide information on various contraceptive methods, such as standard day method (SDM), 2-day method (TDM), lactational amenorrhoea method (LAM), oral contraceptives, condoms, and hormonal injectables. Auxiliary nurses and midwives can also provide education and counseling on all the methods mentioned above, as well as hormone implants and IUDs. Retail outlet operators are authorized to provide contraceptive services in accordance with their clinical qualifications. Nurses and midwives are capable of providing all contraceptive services, but further research is needed for tubal ligation and vasectomy. Allowing other cadres to perform routine tasks restricted to higher-cadre clinicians frees up their time to use their specialized skills for more critical clinical interventions.

With a projected shortfall of 19 million health workers by 2030, mostly in low- and middle-income countries [8], scaling up task-sharing for FP has the potential to expand access and uptake of effective contraceptive methods. Though over 60% of countries have a national policy or guideline on task-sharing [9], the evidence on if and how the task-sharing has been scaled up and sustained in national programs has not been systematically reviewed. Scale-up is defined as “deliberate efforts to increase the impact of successfully tested health innovations to benefit more people and foster policy and program development on a lasting basis” [10] and sustainability (the extent to which an intervention is maintained or institutionalized in a given setting; also known as maintenance, or continuation) [11]. Evidence on the implementation strategies, facilitators, and barriers to scaling up and sustaining task-sharing could inform financing, implementation approaches, and technical assistance of national and global FP task-sharing programs.

### Objective

This study aims to describe and assess the quality of the evidence on implementation strategies, facilitators, and barriers to scaling up and sustaining task-sharing in FP and the outcome of the scale-up/sustainability interventions.

#### Specific questions

- To identify, appraise, and synthesize evidence regarding the approaches or strategies for scaling up and/or sustainability of task-sharing in family planning.
- To identify, appraise, and synthesize evidence on the facilitators and barriers to scaling up and/or sustainability of task-sharing in family planning.
- To identify, appraise, and synthesize evidence on the outcomes of scaling up and/or sustainability of task-sharing in family planning.

## Methods

This systematic review protocol was developed using the Preferred Reporting Items for Systematic Review and Meta-Analysis (PRISMA) Protocols 2015 statement (PRISMA-P) appendix 1 [12], with additional guidance from the guidelines for systematic searches [13], the PRISMA 2020 statement [14], and conducting mixed-methods systematic reviews [15–17]. We registered this protocol in the PROSPERO registry (registration number: CRD42022339885).

### Inclusion criteria

#### Population

**Fig. 1:**
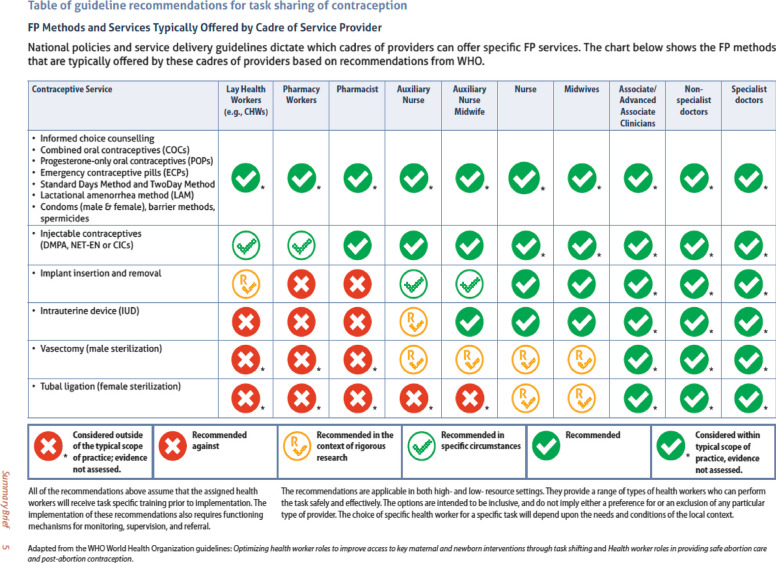
Family planning methods and services typically offered by a cadre of service providers. Geneva: World Health Organization; 2022. License: CC BY-NC-SA 3.0 IGO

The health worker cadres to be considered in this systematic review and their definitions are as described in the WHO brief on task-sharing to improve access to family planning/contraception, namely specialist doctors, non-specialist doctors, advanced associates, and associate clinicians, midwives, nurses, auxiliary nurse midwives (ANM) and auxiliary nurses, doctors of complementary systems of medicine (mainly in South Asia), pharmacists, and pharmacy workers (Fig. 1) [2, 5, 6]. Other groups involved in task-sharing include lay health workers and self-care [2]. All languages, settings, or context, including service provision outlets such as drug shops, pharmacies, and other retail outlets, will be included.

#### Phenomenon of interest

Although task-shifting and task-sharing are slightly different, both are approaches to optimize the limited health workforce; thus, this study adopts the broader term task-sharing for collaborative measures among cadres to optimize health [2, 7]. Task-sharing refers to expanding health cadres who can appropriately deliver health services, not the removal, delegation, or rational distribution from one cadre to another, termed task-shifting [2, 7]. Task-sharing expands cadres that perform all or components of a clinical task hitherto restricted to higher-level cadres among teams of different cadres of health workers [2, 7]. We made a pragmatic decision to limit contraception or FP services eligible for task-sharing and scale-up/sustainability to those recommended by the WHO [2] and to ensure a feasible project comprising relevant services (Fig. 1).

#### Type of intervention

A publication or article is eligible if it reports implementation strategies, facilitators, or barriers to scaling up and sustainability task-sharing in FP/contraception or the outcomes of the scale-up and sustainability interventions. According to the Cochrane Effective Practice and Organization of Care (EPOC) taxonomy of the health system framework, implementation strategies are interventions designed to bring about changes in healthcare organizations, the behavior of healthcare professionals, or the use of health services by healthcare recipients [18]. The change desired in this review is scaling up task-sharing in FP. Literature reporting programs starting with strategic planning for scale-up/sustainability of task-sharing in FP will be included. Similar to previous systematic reviews [19, 20], we will refer to factors that may enable or impede the scale-up/sustainability of task-sharing as facilitators and barriers, respectively. This includes the perceived impact, experiences, and perceptions of these factors as described in qualitative studies.

#### Outcome

Outcomes of scaling up task-sharing in FP would be as defined by Proctor et al. [11]. These include implementation outcomes (acceptability, adoption, appropriateness, costs, feasibility, fidelity, penetration, or sustainability), service outcomes (efficiency, safety, effectiveness, equity, patient-centeredness, or timeliness), or patient outcomes (satisfaction, function, or symptomatology).

#### Type of studies

Any qualitative research, randomized controlled trials, non-randomized studies, quantitative descriptive studies, and mixed-methods studies published in peer-reviewed journals or reports in the gray literature. According to the Mixed Methods Appraisal Tool (MMAT), [21–25] this review will categorize a study as follows:

1. Qualitative research, if it involves common qualitative research approaches, e.g., ethnography, phenomenology, narrative research, grounded theory, case study, and qualitative description, i.e., no specific methodology, but a qualitative data collection and analysis.
2. Randomized controlled trials if participants are randomly assigned to intervention or control groups.
3. Non-randomized studies, if they estimate the effectiveness of an intervention or study other exposures without using randomization, such as non-randomized controlled trials, cohort studies, case–control studies, and cross-sectional analytic studies.
4. Quantitative descriptive studies, if they describe the existing distribution of variables, such as incidence or prevalence studies without comparison groups, surveys, case series, and case reports.
5. Mixed-methods studies, if they use a combination of qualitative and quantitative methods.

### Exclusion criteria

Studies would be excluded if they focused entirely on (1) programs restricted to pilot testing or roll out of task-sharing in FP without scale-up or sustainability components, (2) scale-up/sustainability of FP services that are not recommended by WHO for task-sharing, (3) implementation science theoretical and conceptual development, and (4) contraceptive methods or adverse outcomes. Clinical trial protocols will also be excluded from this review. In the case of duplicate data such as a project or country data reported in multiple studies, an article or project report with the most robust data in terms of recency, quality, and completeness will be prioritized. The other articles or reports will be excluded unless they contain additional information.

Abstracts, editorials, opinion pieces, letters, guidelines, and review articles, including systematic and scoping reviews, are ineligible because this review’s search strategy aims to map task-sharing in FP scale-up/sustainability interventions from their source published articles and gray literature project reports. However, relevant reviews will be explored to determine if any of its primary studies meet this systematic review’s inclusion criteria.

### Information sources and search strategy

The following databases would be searched with no language or date limits: Cumulative Index to Nursing and Allied Health Literature (CINAHL; EBSCOhost), EMBASE (OvidSP), Global Health (OvidSP), MEDLINE (OvidSP), and Scopus (www.scopus.com). Relevant thesaurus headings for “family planning or contraceptive methods” and “task-sharing” would be used, along with free-text search strings constructed for the title or abstract fields. The search terms are adaptations of search strategies of previous systematic reviews on task-sharing [26, 27] and contraception: [28]: The details of the search strategies are provided in Appendix 2.

Gray literature will be identified by searching Google and the websites of organizations, networks, and collaborations working on task-sharing for FP. Additionally, requests would be posted via online networks and listservs for academics, researchers, funders, policymakers, and implementers of programs on task-sharing in FP, such as the WHO IBP Network (https://ibpnetwork.org/) and CoreGroup-Reproductive, Maternal, Newborn, Child and Adolescent Health, and Health Systems Working Groups (https://coregroup.org/our-work/working-groups/#1502865240907-2c473617-a151). Similar keywords for “family planning or contraceptive methods” and “task-sharing” would be used in the gray literature search. The first 100 search results would be reviewed on websites with multiple pages.

The reference lists of all eligible studies would be manually searched for relevant publications. The search strategy would be peer-reviewed using the Peer Review of Electronic Search Strategies (PRESS) guideline [29]. Search results from the different databases would be merged in the Covidence systematic review application to facilitate deduplication, and data would be chatted in Microsoft Excel.

### Data management

#### Selection of studies

After removing duplicates, the search results will first be screened by their titles and abstracts for eligible studies using the inclusion and exclusion criteria. Then, selected full-text publications will be subjected to full eligibility screening. The reason for exclusion at each screening stage will be documented. Search results and included or excluded studies will be summarized using a PRISMA flow diagram. “Google Translate” would be employed to screen titles and abstracts that are not in the English language, and advisers with appropriate language skills would be used for full-text screening. Two independent reviewers would screen and select publications, and disagreements will be resolved by consensus between the reviewers or by a discussion with the coinvestigator team if an agreement cannot be reached.

#### Data extraction

Two independent reviewers will extract data from each study using a structured pre-tested form. In the case of disagreements, a consensus will be reached by discussion between the reviewers or with the coinvestigator team if a consensus cannot be reached. Advisers with appropriate language skills would be employed to extract data from studies not in English. This review will combine data from multiple reports from the same study or project. The information extracted will include the following:

- Author(s)
- Year of publication
- Journal or other types of publications
- Time of data collection (years) or data sources
- Country(ies)
- Objective of the study
- Study design and analysis method
- Targeted population(s)
- Implementation strategies and barriers to scaling up task-sharing for FP
- Outcomes, recommendations, and lessons learned from interventions
- Any other relevant extraction topic

#### Quality assessment

Unlike other quality appraisal tools restricted to specific study designs, thereby necessitating a multiplicity of quality assessment tools while conducting a mixed-methods review, the Mixed Methods Appraisal Tool (MMAT), [21–25] was developed and validated for evaluating different types of studies. It allows for assessing the methodological quality of qualitative research, randomized controlled trials, non-randomized studies, quantitative descriptive studies, and mixed-methods studies and will be used in this study. Two independent reviewers would determine the quality of studies, and disagreements will be resolved by consensus between the reviewers or by a discussion with the coinvestigator team if an agreement cannot be reached.

#### Data analysis and synthesis

This review will describe the evidence on implementation strategies, facilitators, and barriers to scaling up task-sharing FP and the outcome of these interventions. A narrative data-based convergent synthesis will be used, whereby all qualitative, quantitative, and mixed-methods data will be integrated into one dataset [17].

We anticipate a preponderance of qualitative or descriptive data and limited quantitative data as a result of the objectives of this systematic review. Therefore, all quantitative data will be transformed into qualitative data (i.e., themes, categories, or narratives) assembled and integrated into a single dataset alongside the qualitative data [15, 17]. Furthermore, in contrast to quantizing data (i.e., transforming qualitative to quantitative data), the JBI guidelines advise qualitizing data because it is less error-prone [15].

Thereafter, data will be analyzed with an a priori framework [30]. This would involve mapping the implementation strategies, facilitators, and barriers to scaling up and sustaining task-sharing for FP in each eligible publication using the EPOC framework [18]. The EPOC taxonomy covers four health domains: healthcare delivery arrangements, financial arrangements, governance arrangements, and implementation strategies, and the decision for the framework is based on practical experience of the comprehensive synthesis with the tool in a recent review on scale-up strategies for self-administered depot medroxyprogesterone acetate subcutaneous injectable contraception [31]. If applicable, themes would be developed for data that cannot be mapped with this study’s frameworks, and the absence of data in any theme will be noted.

The type of scale-up will also be described in terms of vertical scaling-up, i.e., institutionalization through policy, political, legal, budgetary, or other health systems change or horizontal scaling-up which refers to expansion or replication [10]. Importantly, vertical scale-up provides insight into sustainability. Other outcomes of the implementation strategies to scale up and sustain task-sharing for FP in terms of implementation, service, and client outcomes are as defined by Proctor et al. [11] will also be described. We expect a manageable number of studies. However, if there is an indication that excessive data is likely to compromise synthesis after assessing data richness, we may select a sample of the studies for synthesis [32]. In the case of a package of interventions, the implementation strategies, facilitators, and barriers for the combined intervention will be described. Also, when a factor is reported as both a facilitator and a barrier in different studies, the dominant direction based on a vote counting of articles will be reported. A comment will be added on the possibility that such a factor could act in the reverse direction.

Due to the qualitative synthesis of the data, the quality of evidence will be assessed using the GRADE-CERQual approach [33]. This approach considers four domains: methodological limitations, coherence, adequacy, and relevance. Two reviewers will independently evaluate methodological limitations using the Mixed Methods Appraisal Tool. Disagreements will be resolved by consensus between the reviewers or by a discussion with the coinvestigator team if an agreement cannot be reached. Notwithstanding their quality, all studies that meet this review’s inclusion criteria will be included in the data analysis and synthesis. However, where a theme has high- and low-quality evidence, a sensitivity analysis for high-quality studies may be conducted.

Two reviewers would jointly assess the other three domains, and an overall assessment of the confidence of the evidence will be assigned based on the four domains [34]. In cases of serious concerns with the quality of the evidence, the confidence in the evidence may be downgraded.

### Ethics, patient and public involvement, and dissemination

This research will use publicly available published data; thus, an ethics committee review is not required. Patients or the public were not involved in the design, or conduct, or reporting of this systematic review. However, the research findings will be disseminated in a peer-reviewed journal.

## Discussion and conclusion

To our knowledge, this systematic review of implementation strategies, facilitators, and barriers to scaling up and sustaining task-sharing in family planning is the first in this area. It is based on recent methodological guidelines and will synthesize evidence from different study designs, including quantitative, qualitative, and mixed-methods studies. Thus, we anticipate heterogeneity due to the wide range of study designs and task-sharing concepts in peer-reviewed publications and the gray literature. Nonetheless, this study’s convergent synthesis will integrate all data into one qualitative dataset and assess the quality of evidence with the GRADE-CERQual guideline. This approach can help generate relevant recommendations to support interventions to scale up and sustain task-sharing in family planning. Google Translate’s accuracy in translating narrative and procedural text may not be perfect, which could be a potential limitation.

## Data Availability

Not applicable.

## Appendix 1

**Table 1:** PRISMA-P (Preferred Reporting Items for Systematic Review and Meta-Analysis Protocols) 2015 checklist: recommended items to address in a systematic review protocol

Section and topic	Item No	Checklist item	
Administrative information			
Title			
Identification	1a	Identify the report as a protocol of a systematic review	1
Update	1b	If the protocol is for an update of a previous systematic review, identify as such	NA
Registration	2	If registered, provide the name of the registry (such as PROSPERO) and registration number	3, 5
Authors			
Contact	3a	Provide the name, institutional affiliation, and e-mail address of all protocol authors; provide the physical mailing address of the corresponding author	1
Contributions	3b	Describe the contributions of protocol authors and identify the guarantor of the review	15
Amendments	4	If the protocol represents an amendment of a previously completed or published protocol, identify as such and list changes; otherwise, state a plan for documenting important protocol amendments	NA
Support:			
Sources	5a	Indicate sources of financial or other support for the review	15
Sponsor	5b	Provide the name for the review funder and/or sponsor	15
Role of sponsor or funder	5c	Describe roles of funder(s), sponsor(s), and/or institution(s), if any, in developing the protocol	15
Introduction			
Rationale	6	Describe the rationale for the review in the context of what is already known	3–5
Objectives	7	Provide an explicit statement of the question(s) the review will address with reference to participants, interventions, comparators, and outcomes (PICO)	5
Methods			
Eligibility criteria	8	Specify the study characteristics (such as PICO, study design, setting, time frame) and report characteristics (such as years considered, language, publication status) to be used as criteria for eligibility for the review	5.9
Information sources	9	Describe all intended information sources (such as electronic databases, contact with study authors, trial registers, or other gray literature sources) with planned dates of coverage	9–10
Search strategy	10	Present draft of search strategy to be used for at least one electronic database, including planned limits, such that it could be repeated	9–10
Study records			
Data management	11a	Describe the mechanism(s) that will be used to manage records and data throughout the review	10–14
Selection process	11b	State the process that will be used for selecting studies (such as two independent reviewers) through each phase of the review (that is, screening, eligibility, and inclusion in meta-analysis)	10
Data collection process	11c	Describe the planned method of extracting data from reports (such as piloting forms, done independently, in duplicate), any processes for obtaining and confirming data from investigators	10–11
Data items	12	List and define all variables for which data will be sought (such as PICO items, funding sources), any pre-planned data assumptions and simplifications	10–11
Outcomes and prioritization	13	List and define all outcomes for which data will be sought, including prioritization of main and additional outcomes, with rationale	6
Risk of bias in individual studies	14	Describe anticipated methods for assessing the risk of bias of individual studies, including whether this will be done at the outcome or study level, or both; state how this information will be used in data synthesis	11–12
Data synthesis	15a	Describe the criteria under which study data will be quantitatively synthesized	12–14
	15b	If data are appropriate for quantitative synthesis, describe planned summary measures, methods of handling data, and methods of combining data from studies, including any planned exploration of consistency (such as *I*^2^, Kendall’s *τ*)	12–14
	15c	Describe any proposed additional analyses (such as sensitivity or subgroup analyses, meta-regression)	12–14
	15d	If quantitative synthesis is not appropriate, describe the type of summary planned	12–14
Meta-bias(es)	16	Specify any planned assessment of meta-bias(es) (such as publication bias across studies, selective reporting within studies)	12–14
Confidence in cumulative evidence	17	Describe how the strength of the body of evidence will be assessed (such as GRADE)	13–14

It is strongly recommended that this checklist be read in conjunction with the PRISMA-P Explanation and Elaboration (cite when available) for important clarification on the items. Amendments to a review protocol should be tracked and dated. The copyright for PRISMA-P (including checklist) is held by the PRISMA-P Group and is distributed under a Creative Commons Attribution Licence 4.0 [9]

## Appendix 2

### Ovid MEDLINE® Daily and Ovid MEDLINE®) 1946 to present search strategy

1. (contracepti* or family planning or birth control or depo?-medroxyprogesterone or depo?Medroxyprogesterone or Depo-Provera or Sayana Press or IUD or IUDS or IUS or intra?uterine device* or intra?uterine system* or cervical cap* or vaginal diaphragm* or vaginal ring* or implanon or jadelle or norplant* or sterili?ation or vasectomy or Combined oral contraceptive* or COCs or Progesterone-only oral contraceptive* or POPs or Emergency contraceptive* or ECPs or Standard Days Method or Two Day Method or Lactational amenorrhea method or LAM or Condoms* or barrier method* or spermicide* or tubal ligation).ti,ab. or contraception/ or contraceptive device/ or contraceptive agent/ or family planning/
2. exp personnel shortage/ or (shortage$1 adj5 doctor$1).mp. or (shortage$1 adj5 physician$1).mp. or (shortage$1 adj5 trained adj5 personnel).mp. or (shortage$1 adj5 health adj5 workforce).mp. or (shortage$1 adj5 health adj5 worker$1).mp. or (shortage$1 adj5 health adj5 provider$1).mp. or (task$1 adj5 shift$).mp. or nurse led.mp. or non$1physician clinician$1.mp. or non$1physician health$ worker$1.mp. or primary health care nurs$.mp. or (role adj5 nurs$).mp. or exp community health nursing/ or exp health auxiliary/ or community health$ worker$1.mp. or community health cent$.mp. or lay health$ worker$1.mp. or community health$ aide$1.mp. or (community adj2 health adj5 worker$1).mp. or extended scope practi$.mp. or (role adj3 enhance$).mp. or (substitute$ adj10 physician$1).mp. or (substitute$ adj10 doctor$1).mp. or (substitute$ adj10 nurse$1).mp. or (delegat$ adj10 physician$1).mp. or (delegat$ adj10 doctor$1).mp. or (delegat$ adj10 nurse$1).mp.
